# Development of within-herd immunity and long-term persistence of antibodies against Schmallenberg virus in naturally infected cattle

**DOI:** 10.1186/s12917-018-1702-y

**Published:** 2018-11-26

**Authors:** Kerstin Wernike, Mark Holsteg, Kevin P. Szillat, Martin Beer

**Affiliations:** 1 Institute of Diagnostic Virology, Friedrich-Loeffler-Institut, Greifswald - Insel Riems, Germany; 2 Chamber of Agriculture for North Rhine-Westphalia, Bovine Health Service, Haus Riswick, Kleve, Germany

**Keywords:** Peribunyavirus, Immunity, Intra-herd prevalence, Antibody persistence, Serology, Epidemiology

## Abstract

**Background:**

In 2011, the teratogenic, insect-transmitted Schmallenberg virus (SBV) emerged at the German/Dutch border region and subsequently spread rapidly throughout the European continent. In cattle, one of the major target species of SBV, first antibodies are detectable between one and three weeks after infection, but the duration of humoral immunity is unknown. To assess the course of immunity in individual animals and the development of the within-herd seroprevalence, cattle kept in a German farm with a herd size of about 300 lactating animals were annually blood sampled between December 2011 and December 2017 and tested for the presence of SBV-specific antibodies.

**Results:**

During the monitored period, the within-herd seroprevalence declined from 74.92% in 2011 to 39.93% in 2015 and, thereafter, slightly increased to 49.53% in 2016 and 48.44% in 2017. From the animals that were tested in 2014 and 2015 for the first time (between 24 and 35 months of age) only 14.77% and 7.45%, respectively, scored positive. Thereafter, the seropositivity rate of this age group rose markedly to 58.04% in 2016 and 48.10% in 2017 indicating a circulation of SBV. Twenty-three individual animals were consistently sampled once per year between 2011 and 2017 after the respective insect vector season, 17 of them tested positive at the first sampling. Fourteen animals were still seropositive in December 2017, while three cattle (17.65%) became seronegative.

**Conclusions:**

The regular re-emergence of SBV in Central Europe is a result of decreasing herd immunity caused by the replacement of animals by seronegative youngstock rather than of a drop of antibody levels in previously infected individual animals. The consequences of the overall decline in herd seroprevalence may be increasing virus circulation and more cases of fetal malformation caused by infection of naïve dams during gestation.

## Background

In 2011, an unidentified disease of cattle associated with fever, diarrhea and decreased milk production was reported in Germany and the Netherlands. The causative agent, a member of the Simbu serogroup within the family *Peribunyaviridae*, was eventually identified and named Schmallenberg virus (SBV) [1]. Clinical signs of an SBV infection are restricted to none or mild and transient symptoms in adult animals. However, an infection of naïve ruminants during a critical phase of pregnancy may lead to severe congenital abnormalities, abortion or stillbirth [2].

Like other Simbu serogroup viruses, SBV is primarily transmitted by *Culicoides* biting midges [3–5], while direct transmission between animals via the oral route is highly unlikely [6]. For the spread of potentially infected *Culicoides* midges over long distances, wind seems to play a relevant role [7], since several studies have linked wind movement to the spread of *Culicoides*-born viral diseases [8–10].

The mammalian hosts comprise cattle, sheep, and goats as well as various wild and captive ruminants and some further ungulates and zoo animals [11–16].

In domestic ruminants, SBV-specific antibodies are induced during the first three weeks post infection [6, 17–19] and provide immunity against re-infection [6]. However, the duration of protection remains to be clarified, especially since a previously acquired herd immunity may play an important role in the cyclic re-emergence of the virus, which was observed in Central Europe during recent years [20–22]. Previous studies showed that SBV-specific antibodies are present in the majority of adult cattle for at least two to three years after a natural infection [23–25]. However, about 10% of animals became seronegative within three years [25]. This has raised doubts as to whether the acquired immunity persists for life.

In order to assess the course of immunity in individual animals and the development of the within-herd seroprevalence, cattle kept in a German farm located in the area initially affected most severely by SBV were regularly sampled over a period of six years and tested for the presence of SBV-specific antibodies.

## Results

### Development of within-herd seroprevalence and indications for SBV re-emergence

**Fig. 1:**
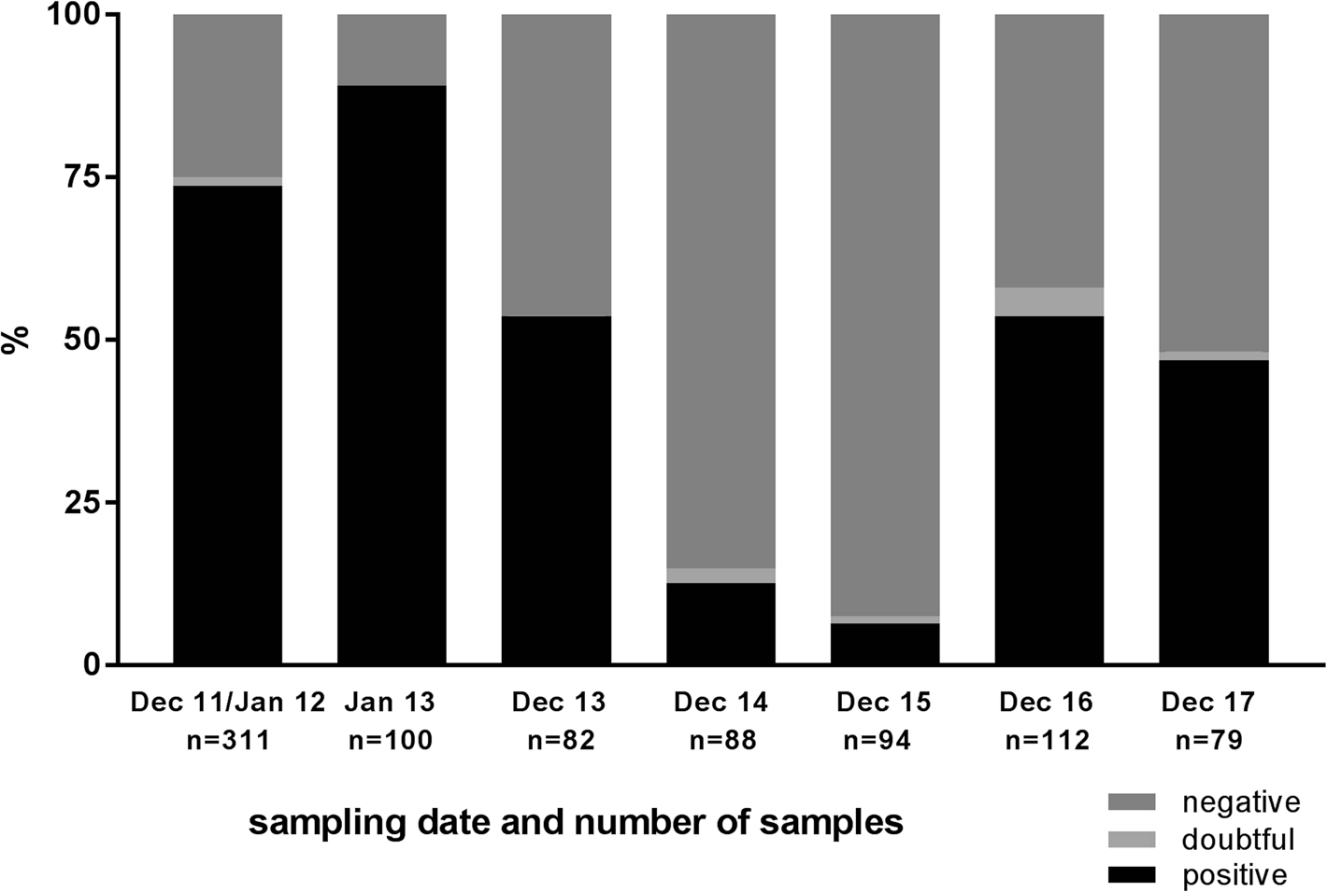
Percentage of anti-SBV antibody positive, doubtful and negative animals among cattle that were sampled for the first time in the respective year. In the December 2011/January 2012 sampling, every animal older than 24 months was included, while thereafter only animals between 24 and 35 months of age were sampled for the first time

A private dairy cattle farm, located in the German federal state North Rhine-Westphalia, was monitored between 2011 and 2017 after the respective insect vector seasons, i.e. in the winter months December or January. The animals included in the study were kept indoors year-round, but all animals younger than 24 months are kept outside. During the monitored period, the within-herd seroprevalence measured by a commercially available SBV antibody ELISA declined from 74.92% in 2011 and 83.08% in 2012 [25] to 39.93% in 2015 and, thereafter, slightly increased to 49.53% in 2016 and 48.44% in 2017. However, the seropositivity rate of animals that were tested for the first time in the respective year (between 24 and 35 months of age) rose drastically from 14.77% in 2014 and 7.45% in 2015 to 58.04% in 2016 and 48.10% in 2017 (Fig. 1).

**Fig. 2:**
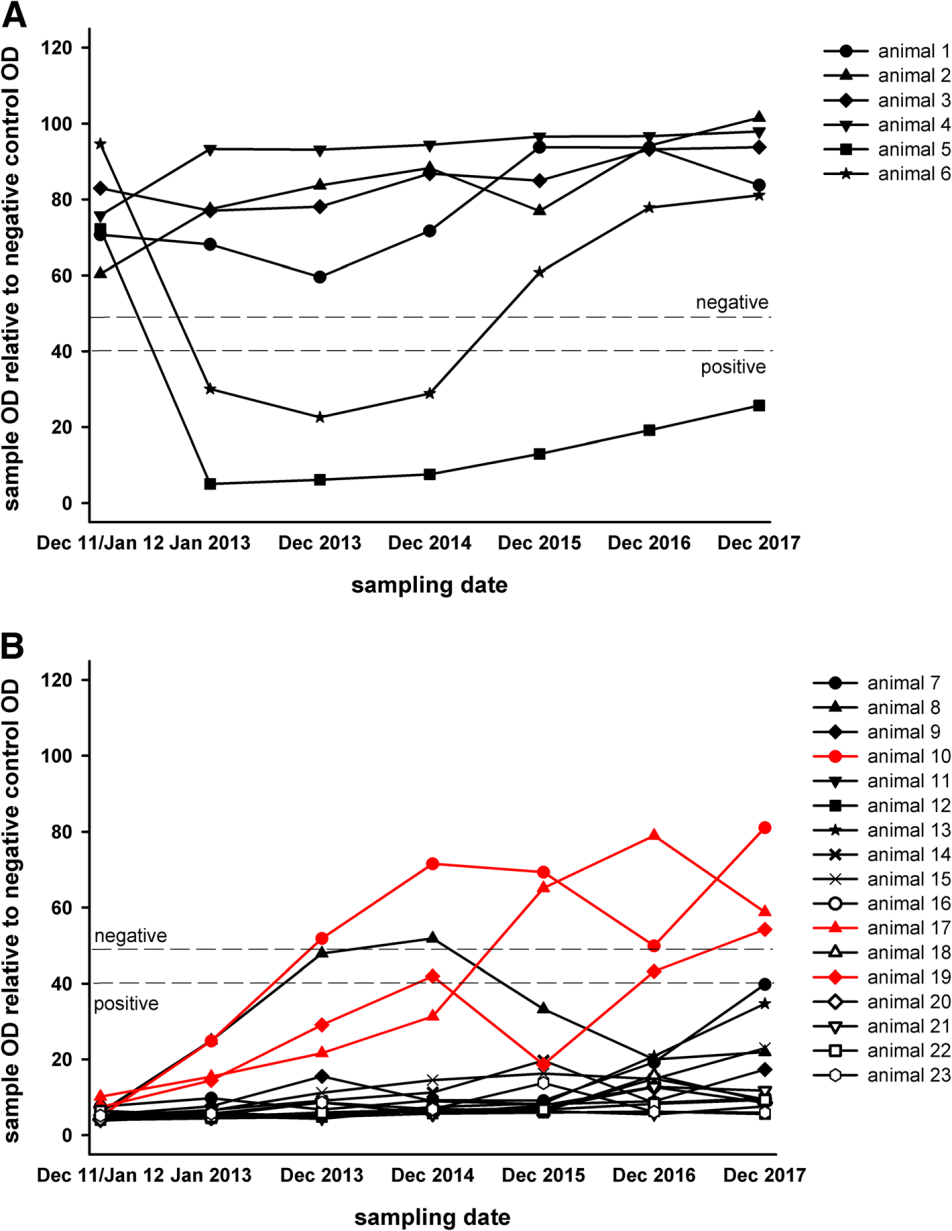
SBV antibody ELISA results of all animals that were consistently sampled once per year between December 2011/January 2012 and December 2017. In figure panel A animals negative at the first sampling time point are shown, while in figure panel B animals that were seropositive at the first sampling are depicted. Animals that tested positive in December 2011/January 2012, but seronegative in December 2017 are marked in red

A total of 23 animals were annually sampled between 2011 and 2017, six of them scored negative in the SBV-ELISA after the 2011 vector season (animal numbers 1–6, Fig. 2a), two of them seroconverted in 2012 (animals 5 and 6, Fig. 2a) and the remaining animals stayed seronegative until the end of the study. Of the other housed, seronegative animals (> 35 months of age), none seroconverted between the 2013 and 2014 and between the 2014 and 2015 sampling dates, but some of these cattle developed SBV-specific antibodies between December 2015 and December 2016 (Table 1). From 12 cattle that tested negative in 2013, five seroconverted in 2016, while from 26 initially in the year 2014 seronegative animals seven tested positive by ELISA in the year 2016, and of the additional 62 cattle seronegative in 2015, one seroconverted in 2016 (Table 1). No further seroconversions were detected between 2016 and 2017 (Table 1).

**Table 1:** Number and status of animals tested for the presence of SBV-specific antibodies at every sampling time point

	number of samples (positive/doubtful/negative)						
status at first sampling	Dec 11/Jan 12	Jan 13	Dec 13	Dec 14	Dec 15	Dec 16	Dec 17
Positive	17/0/0	17/0/0	15/1/1	14/1/2	15/0/2	14/2/1	14/0/3
Negative	0/0/6	2/0/4	2/0/4	2/0/4	1/0/5	1/0/5	1/0/5
Positive		15/0/0	14/1/0	14/1/0	13/0/2	11/1/3	11/0/4
Negative		0/0/0	0/0/0	0/0/0	0/0/0	0/0/0	0/0/0
Positive			13/0/0	13/0/0	11/1/1	11/0/2	9/2/2
Negative			0/0/12	0/0/12	0/0/12	5/0/7	5/0/7
Positive				2/0/0	1/0/1	2/0/0	1/0/1
Negative				0/0/26	0/0/26	7/0/19	7/0/19
Positive					0/0/0	0/0/0	0/0/0
Negative					0/0/62	1/0/61	1/0/61
Positive						29/2/0	28/1/2
Negative						0/0/35	0/0/35
First sampled in 2017 (animals < 36 months of age)							37/1/41
> 36 months of age in 2017, but not consistently sampled in previous years							14/2/6

### Long-term persistence of anti-SBV antibodies

Seventeen of the 23 animals that were annually sampled between 2011 and 2017 tested positive in the SBV antibody ELISA at the first sampling in December 2011/January 2012; 13 of them (76.47%) remained seropositive until December 2017, while three animals (17.65%) became seronegative (Fig. 2b). One animal (number 8 in Fig. 2b) tested positive in January 2013, scored ELISA-doubtful in December 2013, tested negative in December 2014, and scored positive again from 2015 onwards.

## Discussion

After its emergence in 2011 in the Dutch/German border region, SBV spread very rapidly throughout the European continent [26]. In the following years, the virus further spread to previously unaffected regions [27–32], but also repeatedly re-appeared in the center of the initial epidemic [20–22].

In the present study, indications for the re-emergence of SBV in an individual cattle herd were found. A considerable increase of the seropositivity rate was observed in animals that were tested for the first time in 2016 or 2017 compared to animals firstly tested in 2014 and 2015 indicating a re-circulation of SBV in that particular cattle herd leading to seroconversion of naïve youngstock. The age of the animals ranged from 24 to 35 months at the respective first sampling. Since all animals younger than 24 months are kept outside in the monitored herd and grazing increases the risk of an SBV infection for cattle compared to being housed in stables [33], the animals were most likely infected in their first two years of life, i.e. in the 2015, 2016 and/or 2017 vector seasons. But as only a small number of animals (7/94) sampled for the first time in December 2015 was SBV seropositive, large scale circulation of SBV in this year seems unlikely. Furthermore, none of the seronegative, older, housed animals seroconverted in 2015, but some of these cattle developed SBV-specific antibodies between December 2015 and December 2016. This observation and the fact that no further seroconversions were detected between 2016 and 2017 suggests virus circulation in the cattle herd in the 2016 vector season, which is in line with previously reported SBV-detections in North-Rhine Westphalia and further German federal states in that year [20]. Such patterns of cyclic re-circulation in a given area as currently seen in the case of SBV have also been described for other Simbu serogroup viruses. There are for example regular epidemics of Akabane virus (AKAV), Aino virus and Peaton virus in Japan [34–36] or of AKAV in Australia [37].

Regarding the long-term persistence of virus-specific antibodies, however, little information is available for these Simbu serogroup viruses, since the booster effect caused by re-infections of animals kept in endemic areas hampers attempts to measure the development of antibody levels in naturally infected, commercial cattle. For AKAV, it has been described that specific antibodies persist for at least two years [38]. The same holds true for SBV, and specific antibodies are detectable in the majority of cattle for at least two to three years [23–25].

In the present study, naturally infected cattle were monitored over a period of six years and only three out of 17 animals became seronegative in this time frame, while anti-SBV antibodies were still measurable after six years in the remaining 14 cattle. One of these animals tested negative at one sampling date (very close to the cut-off value) and again clearly positive at subsequent time points which might be caused by mixing-up animals during sampling or a false-negative test result or by a re-infection of that animal. A re-infection of all animals, however, is very unlikely, as the relatively low rate of seroconversions measured in 2016 in adult animals that are kept indoors compared to the higher rate seen in the youngstock, which is kept outside, further confirms that an SBV infection of housed animals is less likely than that of grazing cattle as was already previously reported [33]. Consequently, most of the seropositive housed animals, which were tested annually between 2011 and 2017, presumably were not re-infected and the measurable antibodies represent a specific humoral immunity acquired during their first infection in the 2011 or 2012 vector seasons. Hence, anti-SBV antibodies persist in the majority of cattle for at least six years, if not even lifelong.

## Conclusions

Antibodies acquired following a natural SBV infection were detectable in more than 75% of the cattle monitored in the present study over a period of six years. Therefore, the regular re-emergence of SBV in given areas is more likely a result of decreasing herd immunity caused by replacement of animals by seronegative youngstock, which are susceptible once maternally-derived antibodies have declined at an age of 5 to 6 months [23, 24], than by a drop of antibody levels in previously infected animals. The result of the overall decline in the herd seroprevalence and the following renewed virus circulation may be again more frequent infections of naïve heifers during gestation and, as a consequence the induction of fetal malformations.

## Methods

A private dairy cattle farm, located in the federal state North Rhine-Westphalia, was monitored between 2011 and 2017 after the respective insect vector seasons, i.e. in the winter months December and January. The cows included in the study were kept indoors year-round under routine production conditions, but all animals younger than 24 months are kept outside.

Serum samples of all cows older than 24 months were taken in December 2011 or January 2012, January 2013, December 2013, December 2014 [25], December 2015 (288 animals), December 2016 (317 animals), and December 2017 (320 animals).

From a total of 23 animals, routine diagnostic blood samples were available from every sampling date, the age of these animals ranged in December 2017 from 96 to 160 months.

Serum samples were taken by puncture of the vena coccygea and analyzed by a commercially available SBV antibody ELISA (ID Screen® Schmallenberg virus Competition, ID vet, France) according to the manufacturer’s instructions. For the calculation of the within-herd seroprevalences, doubtful results were considered as positive.

## Abbreviations

AKAV: Akabane virus
SBV: Schmallenberg virus
